# Nitric Oxide Regulates Input Specificity of Long-Term Depression and Context Dependence of Cerebellar Learning

**DOI:** 10.1371/journal.pcbi.0020179

**Published:** 2007-01-12

**Authors:** Hideaki Ogasawara, Tomokazu Doi, Kenji Doya, Mitsuo Kawato

**Affiliations:** 1 ATR Computational Neuroscience Laboratories, Seika, Kyoto, Japan; 2 National Institute of Information and Communications Technology, Seika, Kyoto, Japan; 3 Graduate School of Information Science, Nara Institute of Science and Technology, Takayama, Ikoma, Nara, Japan; 4 Initial Research Project, Okinawa Institute of Science and Technology, Uruma, Okinawa, Japan; University College London, United Kingdom

## Abstract

Recent studies have shown that multiple internal models are acquired in the cerebellum and that these can be switched under a given context of behavior. It has been proposed that long-term depression (LTD) of parallel fiber (PF)–Purkinje cell (PC) synapses forms the cellular basis of cerebellar learning, and that the presynaptically synthesized messenger nitric oxide (NO) is a crucial “gatekeeper” for LTD. Because NO diffuses freely to neighboring synapses, this volume learning is not input-specific and brings into question the biological significance of LTD as the basic mechanism for efficient supervised learning. To better characterize the role of NO in cerebellar learning, we simulated the sequence of electrophysiological and biochemical events in PF–PC LTD by combining established simulation models of the electrophysiology, calcium dynamics, and signaling pathways of the PC. The results demonstrate that the local NO concentration is critical for induction of LTD and for its input specificity. Pre- and postsynaptic coincident firing is not sufficient for a PF–PC synapse to undergo LTD, and LTD is induced only when a sufficient amount of NO is provided by activation of the surrounding PFs. On the other hand, above-adequate levels of activity in nearby PFs cause accumulation of NO, which also allows LTD in neighboring synapses that were not directly stimulated, ruining input specificity. These findings lead us to propose the hypothesis that NO represents the relevance of a given context and enables context-dependent selection of internal models to be updated. We also predict sparse PF activity in vivo because, otherwise, input specificity would be lost.

## Author Summary

The cerebellum is essential for coordinated movements. The skills for executing such movements are acquired in modules of the cerebellum, and the appropriate modules in which to store the skill for a certain movement are selected according to the environment, or the context, where the movement is made. We are interested in the molecular mechanisms that enable context-dependent cerebellar learning. In search of the key molecules, we combined established simulation models of Purkinje cells, the only output neurons in the cerebellar cortex, and constructed a new model. Using computer simulation, we found that nitric oxide is likely to have a pivotal role in context-dependent learning. Our simulation also provides insights into how sparse sensory information is coded in the cerebellar cortex. These findings have led us to propose the experimentally testable hypothesis that the relevance of a given context to learning modules is represented by the concentration of nitric oxide.

## Introduction

Internal models—that is, neural mechanisms for motor planning and control—mimic the input/output characteristics of the motor apparatus or their inverses [1]. Recent studies have shown that multiple internal models are acquired in the cerebellum [2], and that these can be switched under a given context of behavior [3,4], so that, for example, we can walk on stairs or on escalators without losing balance. Contextual information, which consists of various modalities of afferent signals from the entire body and efferent signals from the cerebrum, is transmitted to the cerebellar cortex via mossy fibers (MFs) and parallel fibers (PFs) [5–7]. Contexts may also be processed in the upstream cerebral regions, such as the superior parietal lobe, the occipital lobe, and the middle temporal lobe [4]. The combination of contexts and tasks is thought to enable the cerebellum to acquire and switch between multiple internal models in a context-dependent manner [1,6,7]. Indeed, adaptation to multiple motor tasks is possible when they are presented together with adequate sensory or cognitive cues [8–11]. On the other hand, without these contexts, the motor memory of a previously learned task would be erased by the experience of an opposing task [12]. However, the biological mechanisms of context-dependent learning have yet to be explored.

Long-term depression (LTD) of PF–Purkinje cell (PC) synapses is widely thought to form the cellular basis of cerebellar learning [13], with some controversies regarding its computational roles and the participation of other types of plasticity [14–17]. The presynaptically synthesized messenger nitric oxide (NO) is a “gatekeeper” to plasticity at these synapses; LTD and long-term potentiation are induced only in its presence [13,18–21]. Because NO diffuses freely to neighboring synapses and affects them [22,23], this volume learning, unlike classical associative learning, is not input-specific and raises questions about the biological significance of LTD as the basic mechanism for efficient supervised learning.

**Figure 1:**
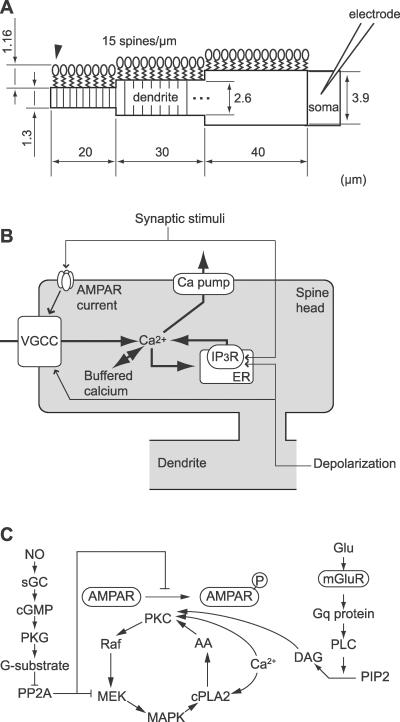
Overview of the Model (A) The electrophysiological structure of the model. Spine necks connect spine heads (arrowhead) to an unbranched dendrite. The right-hand end of the dendrite corresponds to the soma, which is voltage-clamped. Vertical lines indicate compartmentalization of the dendrite. (B) Block diagram of signaling pathways for calcium mobilization in each spine. Thick arrows and thin arrows indicate mobilization of calcium and activation of targets, respectively. (C) Block diagram of AMPAR phosphorylation. Raf is a kinase that phosphorylates and activates MEK. Gq protein is a heterotrimeric guanine nucleotide binding protein that activates PLC. AA, arachidonic acid; DAG, diacylglycerol; PIP2, phosphatidylinositol bisphosphate; PKG, cGMP-dependent protein kinase; PLC, phospholipase C; PP2A, protein phosphatase 2A; sGC, soluble guanylyl cyclase.

To better characterize the role of NO in cerebellar learning, we constructed a new comprehensive model based on established simulation models of the electrophysiology, calcium dynamics, and signaling pathways of the PC [24–26]. It is basically a passive electrical cable representing a PC dendrite (Figure 1A) whose dendritic spines contain ion channels, calcium buffers, calcium pumps (Figure 1B), and a biochemical reaction network (Figure 1C). For our purposes, it was necessary to simulate the whole sequence of events in PF–PC LTD, from stimulation of PF–PC synapses coupled with depolarization, diffusion of NO, and an increase of calcium concentration in the spine ([Ca^2+^]_spine_), to activation of the intracellular signaling cascade and phosphorylation of alpha-amino-3-hydroxy-5-methyl-4-isoxazole propionate receptors (AMPARs), using a rather complicated model, because cerebellar LTD is a very nonlinear phenomenon [25,26].

Our simulation revealed that a PF–PC synapse undergoes LTD only when more than several other PFs in the vicinity are activated concomitantly. This finding has led us to propose a novel hypothesis of cerebellar learning in which NO enables context-dependent acquisition and updating of internal models; in addition, we suggest an animal experiment to critically test this hypothesis. We also predict that the number of PFs responsible for a certain movement in a certain context needs to be strictly limited, because, otherwise, input specificity would be lost.

## Results

### Outline of the Model

We combined several established models of the PC [24–26] and constructed a new comprehensive model to simulate the sequence of electrophysiological and biochemical events in cerebellar LTD. The electrophysiological part of the model (Figure 1A) is based on a realistic PC model proposed by De Schutter et al. (De Schutter's model) [24] but is greatly simplified. It consists of a 90-μm dendritic cable that is as long electrically as the average PC dendrite [27] and is composed of 30 compartments. The peripheral ten compartments (Figure 1A, left side) are 1.3 μm in diameter and 2 μm in length; the middle ten are 2.6 μm in diameter and 3 μm in length; and the proximal ten (Figure 1A, right side) are 3.9 μm in diameter and 4 μm in length. A total of 1,350 single-compartment spines are connected to the dendrite (15 spines per micrometer of the dendrite length [28,29]) by a spine neck. The electrical potential of each spine and dendritic compartment is expressed in cable equations [30]. Despite considerable alterations to De Schutter's model, our model retains important characteristics of the PC, as expected from the robustness of the original model to any changes made in its parameter values [31]. For example, the input resistance measured at the tip of the dendrite is 129 MΩ, which is on the same order as the values estimated from real PCs (132–511 MΩ) [27].

Stimuli to PF–PC synapses are represented as AMPAR currents into the spines** (** Figure 1B), metabotropic glutamate receptor (mGluR) activation (Figure 1C), and diffusion of presynaptically synthesized NO. We assumed a constant release probability and did not model presynaptic plasticity. The kinetics of NO and calcium are introduced in later subsections. Spillover of glutamate was not considered, because granular layer stimulation and molecular layer stimulation that involve approximately 60 PF–PC synapses each have been reported to produce excitatory postsynaptic currents with almost equal decay time constants [32], suggesting that glutamate spillover has only minimal effects, if any, on the extent of stimulation in our study. Inhibitory interneurons were not simulated.

The biochemical reaction network for LTD (Figure 1C) was derived from a model proposed by Kuroda et al. (Kuroda's model) [26]. According to their study, AMPAR phosphorylation, the key step in expression of cerebellar LTD [13,26], is regulated in the initial phase by Ca^2+^- and diacylglycerol-mediated linear pathways, and is maintained in the intermediate phase by a positive feedback loop pathway that is mediated by mitogen-activated protein kinase (MAPK), cytosolic phospholipase A2 (cPLA2), and protein kinase C (PKC). Indeed, the essential role of this feedback loop in cerebellar LTD has been virtually proved by a series of in vitro pharmacological experiments demonstrating the following: PKC activation results in MAPK activation; MAPK activation results in PKC activation; cPLA2 is activated by MAPK and activates PKC; and prolonged activation of PKC is necessary for induction of LTD [33]. Similar to Kuroda et al., we measured the concentration of phosphorylated AMPARs ([P-AMPAR]) as the output of the simulation.

### Production, Diffusion, and Decay of Nitric Oxide

**Figure 2:**
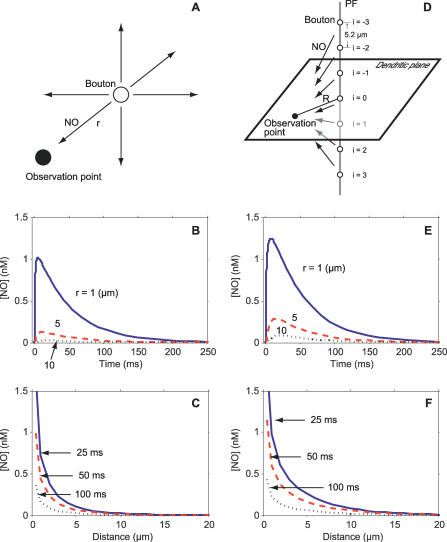
Simulated Concentration of NO Derived from a Single Bouton or from Multiple Boutons on a PF (A–C) NO derived from a single bouton. (A) Activated NOS in a bouton (open circle) produces NO, which diffuses three dimensionally and decays. Only some of the synthesized NO reaches the observation point (closed circle). r*,* distance between the observation point and the NO-producing bouton. (B) [NO] is plotted against time. The blue solid line, red dashed line, and black dotted line indicate [NO] measured at 1 μm, 5 μm, and 10 μm, respectively, from the bouton. (C) [NO] is plotted against distance. The blue solid line, red dashed line, and black dotted line indicate the concentration at 25 ms, 50 ms, and 100 ms, respectively, from stimulation of the bouton. (D–F) NO derived from boutons along a PF. (D) When a PF fires, NO is released from its boutons (open circles) located at 5.2-μm intervals along the PF and diffuses [23,34]. Thus, [NO] measured at the observation point (closed circle) on the dendritic plane at distance R from the PF is the sum of [NO] from each bouton at distance
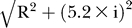
 μm (i = 0, ±1, ±2,...). Of course, NO diffuses from each bouton in all directions, but only diffusion towards the observation point is indicated (arrows) for clarity. (E) [NO] is plotted against time. The blue solid line, red dashed line, and black dotted line represent [NO] at 1 μm, 5 μm, and 10 μm, respectively, from the PF. (F) [NO] is plotted against distance from the PF. The blue solid line, red dashed line, and black dotted line indicate the concentration at 25 ms, 50 ms, and 100 ms, respectively, after stimulation of the PF. [NO] at distances smaller than 0.5 μm are not shown in (C) and (F), because the delta function in Equation 1 (Materials and Methods) made the NO concentration near the site of synthesis dependent on the discretization size of *r* in its numerical solution.

Firing of PFs activates NO synthase (NOS) in their presynaptic terminals [13,18,19,21]. The NO produced by NOS then freely diffuses into the intracellular and extracellular spaces and decays, affecting postsynaptic signaling pathways en route [13,20,21,23]. We modeled the decay and diffusion of NO synthesized at a PF bouton (Figure 2A) and calculated the NO concentration ([NO]) at various distances from the bouton and at various times. Figure 2B is a plot of the time course of NO synthesized in a bouton at various distances from the observation point, and Figure 2C shows its spatial distribution at various time points. [NO] rapidly peaked after activation of NOS, then gradually fell (Figure 2B). The distribution of NO was restricted to within 10 μm or 20 μm of its source (Figure 2C). In reality, however, when a PF fires, NO is not synthesized at a single bouton but at multiple neighboring boutons, which occur every 5.2 μm along the PF [18,19,21,34] (Figure 2D). These boutons also contribute to [NO] at the observation point on the PC dendritic plane, according to their distance away from the observation point; that is, 
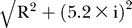
 μm (i = 0, ±1, ±2,...), where R is the distance between the PF and the observation point, and i is the number assigned to a given bouton, with i = 0 for the one on the dendritic plane. Figure 2E and 2F are plots of the time course of PF-derived NO measured at various distances, and of its spatial distribution at various time points, respectively. NO derived from a PF persisted slightly longer than that derived from a bouton. The concentrations of NO derived from a PF at R = 1 μm, 5 μm, and 10 μm returned to 36.8% of their peak values in 64 ms, 72 ms, and 79 ms, respectively (Figure 2E), whereas the concentrations of NO derived from a single bouton at the same distances returned to 36.8% of their peak values in 59 ms, 67 ms, and 75 ms, respectively (Figure 2B). In addition, NO derived from a PF was spatially less restricted than that derived from a single bouton. At 25 ms, 50 ms, and 100 ms after activation of NOS, the ratios of the [NO] derived from a PF at R = 10 μm to that at R = 5 μm were 0.33, 0.35, and 0.35, respectively (Figure 2F), whereas the ratios of the [NO] derived from a bouton at R = 10 μm to that at R = 5 μm were 0.23, 0.24, and 0.24, respectively (Figure 2C). The spatial distribution of NO synthesized in a PF (Figure 2F) quantitatively agrees with the results of a previous slice experiment [35].

### Calcium Kinetics

The calcium kinetics of our model (Figure 1B) are based on De Schutter's model [24] and on a calcium dynamics model of a PF–PC synaptic spine proposed by Doi et al. (Doi's model) [25]. Briefly, calcium ions enter the cytosol of the spine through voltage-gated calcium channels (VGCCs) in the plasma membrane and through inositol 1,4,5-triphosphate (IP_3_) receptors (IP_3_Rs) in the endoplasmic reticulum (ER) [13,25,36], and are sequestered by calcium buffers, taken in by the ER, or pumped out by calcium pumps. Depolarization of the dendrite, whether by AMPAR currents or by propagation from other parts of the dendrite, opens VGCCs. The mGluR–IP_3_R pathway detects the coincidence of PF inputs and depolarization, and activates a large release of calcium from the ER [25]. For simplicity, our model postulates that during the 2 s after each stimulus, a fixed number (5.3 × 10^4^) of calcium ions flow into the stimulated spines (Figure S1), instead of actually simulating the mGluR–IP_3_R pathway [25].

**Figure 3:**
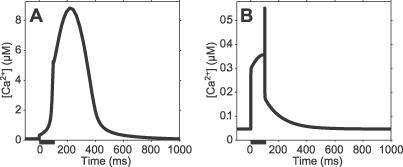
Time Course of [Ca^2+^]_spine_ [Ca^2+^]_spine_ was monitored during and after 100 ms of somatic depolarization (−70 mV to 0 mV, bold bars) in the presence (A) and absence (B) of a conjunctive stimulus to the synapse at time = 0 ms.

To understand the calcium kinetics in our model, we stimulated PF–PC synapses conjunctively with somatic depolarization (100 ms in duration) and monitored [Ca^2+^]_spine_ (Figure 3). [Ca^2+^] reached approximately 9 μM in 200 ms in the spines that were directly stimulated (Figure 3A); [Ca^2+^] increased by a much smaller extent in the neighboring spines that were not directly stimulated (Figure 3B). These findings are quantitatively consistent with the results of previous experiments [36,37]. Although we had expected that stimuli to neighboring synapses would locally depolarize the dendrite, opening VGCCs in the spine and allowing more calcium to enter, the size and waveform of [Ca^2+^]_spine_, either in stimulated synapses or in synapses not directly stimulated, were practically identical and independent of the number of stimulated synapses within the range tested (one to 30).

### Time Course of AMPAR Phosphorylation

**Figure 4:**
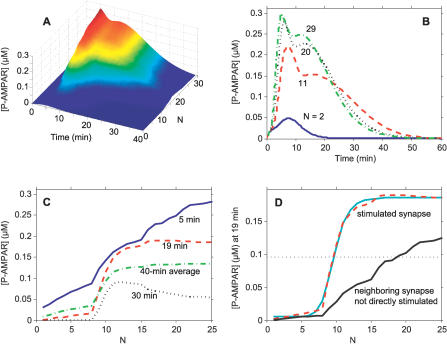
Concentration of Phosphorylated AMPARs at Various Time Points and at Various Numbers of Stimulated Synapses (A) [P-AMPAR] is three-dimensionally plotted against time and N. (B) [P-AMPAR] plotted against time at N = 2 (blue solid line), 11 (red dashed line), 20 (black dotted line), and 29 (green dash-dotted line). (C) [P-AMPAR] plotted against N at time = 5 min (blue solid line), 19 min (red dashed line), and 30 min (black dotted line), and at the 40-min average (green dash-dotted line). (D) [P-AMPAR] measured at 19 min plotted against N in a stimulated synapse (the synapse of interest, red dashed line) or a neighboring unstimulated synapse (black solid line). [P-AMPAR] of the stimulated synapse is superimposed on a sigmoid curve (light blue solid line) at an SF of 9.9 and N_50%_ of 9.7 (see Results). The thin dotted line indicates [P-AMPAR]_50%,19min_.

We then observed the time course of AMPAR phosphorylation after synaptic stimulation paired with somatic depolarization. A synapse 9 μm proximal from the tip of the dendrite (the synapse of interest) was repetitively stimulated at 1 Hz for 5 min together with zero to 29 neighboring synapses (0–20 μm from the tip of the dendrite; Table S1), and the soma was depolarized from −70 mV to 0 mV for 100 ms simultaneously with each synaptic stimulus. NO synthesized not only in the stimulated synapses but also in the other boutons along the same PFs (Figure 2D–2F) was considered. [P-AMPAR] was monitored in the synapse of interest (the synapse 9 μm from the tip) as a measure of LTD induction. Figure 4A is a 3-D graph of [P-AMPAR] plotted against the number of stimulated synapses (N) and time, and Figure 4B shows sections from Figure 4A when N = 2, 11, 20, and 29. When two PF–PC synapses were stimulated, only a small proportion of the AMPARs were phosphorylated, and these were soon dephosphorylated (Figure 4B). This result is in accordance with a slice experiment in which stimulation of a small number of PFs, coupled with depolarization, failed to induce LTD [38]. Application of stimuli to a larger number of synapses led to stronger and more prolonged phosphorylation of AMPARs (11 or more synapses; Figure 4B). At any strength of stimulus, [P-AMPAR] returned to baseline within an hour (Figure 4A and 4B). In reality, however, LTD, which also involves receptor internalization, gene expression, and protein synthesis, is more persistent than the AMPAR phosphorylation modeled here [13,39,40].

### The All-or-None Principle in the Intermediate Phase

Kuroda et al. [26] have pointed out that AMPAR phosphorylation in the intermediate phase, which corresponds to the success of induction of LTD, follows the all-or-none principle. Figure 4C is a graph of simulated [P-AMPAR] plotted against N at 5 min, 19 min, and 30 min, and the 40-min average of [P-AMPAR]. In our model, AMPAR phosphorylation initially occurred in a graded fashion according to N (Figure 4A, ∼5 min; Figure 4C, 5 min), but persisted in an all-or-none fashion in the intermediate phase (Figure 4A, 10–30 min; Figure 4C, 19 min and 30 min). To evaluate the nonlinearity of AMPAR phosphorylation in the intermediate phase, the [P-AMPAR] values at various time points versus N were fitted to a sigmoid curve, [P − AMPAR] = [P − AMPAR] _N = 0 _ + 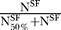 ([P − AMPAR] _N = ∞_ − [P − AMPAR] _N = 0_), where [P-AMPAR] _N = 0_, [P-AMPAR] _N__= ∞_, and N_50%_ are [P-AMPAR] at N = 0, [P-AMPAR] estimated at N = ∞, and N at which [P-AMPAR]_50%_ 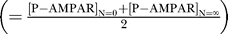 of AMPARs arephosphorylated, respectively. SF stands for the sloping factor, which indicates the nonlinearity of the response. Goodness of fit was quantified by the ratio of the confidence interval of the SF to the SF. [P-AMPAR] best fitted a sigmoid curve at 19 min with a SF of 9.9 (95% confidence interval, 8.7–11.1), indicating strong nonlinearity of AMPAR phosphorylation in the intermediate phase (Figure 4C; see also Figure 4D for [P-AMPAR] at 19 min with the sigmoid curve superimposed). The curve fitting estimated a [P-AMPAR]_N=∞_ of 0.19 μM and an N_50%_ of 9.7. The [P-AMPAR] at other time points, and the 40-min average, fitted only poorly to sigmoid curves. We therefore concluded that the [P-AMPAR] at 19 min best reflects successful induction of LTD.

**Figure 5:**
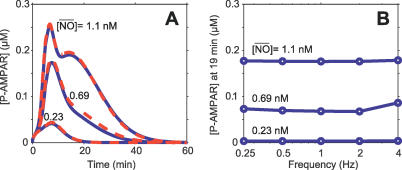
Importance of the Time-Averaged Concentration of NO (A) Time course of AMPAR phosphorylation induced by continuous exposure to NO (red dotted line) or by NO pulses (blue solid line) equivalent in time-averaged concentrations (0.23 nM, 0.69 nM, and 1.1 nM from bottom to top). (B) [P-AMPAR] at 19 min is plotted against the frequencies of NO pulses whose 1-s–averaged concentration was 0.23 nM, 0.69 nM, or 1.1 nM (from bottom to top).

### Synaptic Specificity

LTD has been reported to spread to neighboring PF–PC synapses in vitro when dozens of PFs are stimulated in synchrony with somatic depolarization [38,41,42]. In other words, under such conditions, a submicromolar increase in [Ca^2+^]_spine_ is sufficient for induction of LTD. However, stimulation of a smaller number of PFs, coupled with depolarization, fails to induce LTD, even in stimulated synapses [38]. Furthermore, when the stimulation parameters are varied, stimulated synapses and their neighboring synapses often undergo modification in the same direction—that is, either long-term potentiation or LTD [38]. These findings conflict with the proposed role of cerebellar LTD in supervised learning [13], in which input specificity is taken for granted. We therefore compared AMPAR phosphorylation in a stimulated spine and an adjacent spine to determine whether there is any opportunity for synaptic specificity. Figure 4D plots [P-AMPAR] at 19 min after stimulation in a stimulated synapse and a neighboring synapse 9 μm from the tip of the dendrite against the number of stimulated synapses, N. The slope of [P-AMPAR] in the neighboring synapse was less steep than that in the stimulated synapse, probably because the MAPK-mediated positive feedback loop, which is responsible for the bistability of AMPAR phosphorylation [26], is not fully activated by submicromolar [Ca^2+^]_spine_. At large Ns, however, the slope eventually reached values comparable with those in the stimulated synapse. The slope for the stimulated synapse crossed [P-AMPAR]_50%_ at 19 min ([P-AMPAR]_50%,19min_) when N = 10, whereas that of the neighboring synapse did not cross this value until N reached 19. We therefore concluded that synapse-specific LTD is theoretically possible within a narrow range of N (ten to 18), although experiments have failed to clearly demonstrate input specificity of LTD [38]. Just by adjusting parameters so that LTD would begin to spread through the neighborhood at an N of about 20 (Materials and Methods), our simulation succeeded in demonstrating the failure of LTD at smaller Ns (Figure 4B), which has also been shown experimentally [38].

### The Time-Averaged Concentration of NO, Not Its Actual Waveforms, Is Critical for AMPAR Phosphorylation

Although only a fraction of PFs form electrically connected synapses with a PC [43], other PFs can also affect these synapses by producing NO. PFs convey diverse modalities of signals, and the set of PFs responsible for a certain motion in a certain context does not always fire in exact synchrony. The more out of synchrony they fire, the more blunt the NO waveforms become. Taking the slow time constant of protein phosphatase 2A inactivation [26] (Figure 1C) into account, we hypothesized that [P-AMPAR] would depend not on NO waveforms, but rather on its time-averaged concentration. To test this hypothesis, we compared receptor phosphorylation induced by pulses of NO and that induced by continuous exposure to NO. A spine was stimulated for 5 min, either repetitively at 1 Hz with realistic waveforms of NO (identical in shape to the solid line in Figure 2E and variable in amplitude), or constantly with the equivalent time-averaged concentration of NO. Calcium surges (identical in shape and amplitude to the curve in Figure 3A) were applied concomitantly at 1 Hz. Figure 5A shows the time course of AMPAR phosphorylation induced by constant NO and that induced by equivalent pulses. Their time courses almost coincide, suggesting that constant NO can substitute for equivalent pulses in AMPAR phosphorylation. We then tested NO waves at various frequencies to further confirm the independency of AMPAR phosphorylation from the waveforms of NO. It should be noted that what we examined here is not the frequency of stimuli to PFs, upon which presynaptic NO synthesis is dependent [18,35]. A spine was stimulated for 5 min with NO pluses at a frequency of 0.25 Hz, 0.5 Hz, 1 Hz, 2 Hz, or 4 Hz, and with calcium surges at 1 Hz. The NO pulses were rescaled so that their time-averaged concentrations would be the same. Figure 5B shows [P-AMPAR] at 19 min, the time point when the all-or-none principle of AMPAR phosphorylation is most obvious (Figure 4C), plotted against the frequencies of NO pulses. This figure indicates that the effect of NO on AMPAR phosphorylation is independent of its frequency within the range shown. Because of these findings, we were able to define the concentration of constant NO that would result in [P-AMPAR]_50%,19min_ of phosphorylated AMPARs at 19 min (median effective concentration, or EC_50_). The EC_50_ of NO for a stimulated spine and that for a neighboring spine are 0.41 nM and 0.69 nM, respectively. Introduction of the EC_50_ of NO enabled us to predict PF activity in vivo in a very simple scheme, as described below.

### Distribution of PFs Assigned to a Certain Movement in a Certain Context

A fraction of PFs fire to achieve a certain movement in a certain context [6,7], but their distribution is totally unknown. At one extreme, they might all be concentrated in a small area of a PC dendrite; at the other extreme, they might be evenly distributed in the molecular layer, with only one of them forming an electrically active synapse with the PC (if none of them formed an electrically active synapse, there would be no room for synapse-specific modification linked to that movement). We were able to examine the relationship between PF distribution and AMPAR phosphorylation in a simple scheme in which [NO] is constant throughout stimulation, because AMPAR phosphorylation depends on the time-averaged concentration of NO, and not on its actual waveforms (Figure 5). Synaptic stimulation was represented as NO synthesis, AMPAR current, and mGluR activation, but we were also able to regard neighboring PFs merely as additional sources of NO, because stimuli to them had hardly any effect on [Ca^2+^]_spine_ in the synapse of interest, and because the downstream signaling cascade of mGluR activation was synapse-specific, at least in our model. In other words, it was unnecessary to distinguish the minority of PFs that formed electrical connections with the PC [43] from the other PFs that were not connected to that PC [43]; the only thing that mattered was the number of firing PFs in the neighborhood of the synapse of interest. To make this point obvious, in this section we use “n” instead of “N” to indicate the number of activated PFs.

**Figure 6:**
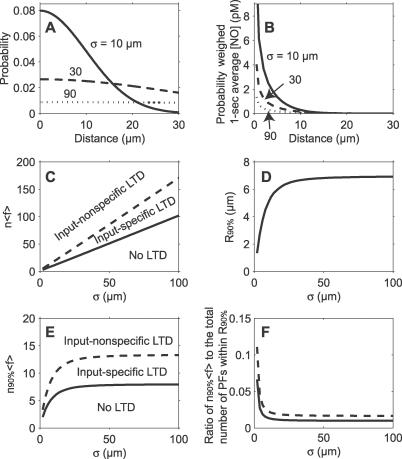
Prediction of PF Activity In Vivo The spatial distribution of the PFs responsible for a certain movement in a certain context was assumed to follow a Gaussian distribution with a standard deviation of *σ*. (A) The occurrence of the PFs responsible for a particular motion in a particular context was assumed to follow a Gaussian distribution,
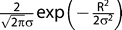
 . *σ* is either 10 μm (solid line), 30 μm (dashed line), or 90 μm (dotted line). (B) The probability-weighed time-averaged NO concentration,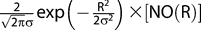 , is plotted against R. *σ* is either 10 μm (solid line), 30 μm (dashed line), or 90 μm (dotted line). (C) Against *σ,* this panel plots the n<f> at which [P-AMPAR]_50%,19min_ of AMPARs in a stimulated synapse (solid line) or in a neighboring unstimulated synapse (dashed line) was phosphorylated at 19 min. (D) R_90%_, which satisfies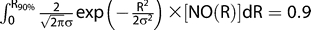 , is plotted against *σ*. (E) n_90%_<f> (see Results for explanation) is plotted against *σ*. Solid line, stimulated synapse; dashed line, neighboring unstimulated synapse. (F) The ratio of n_90%_<f> to the total number of the PFs projecting within R_90%_ μm of the dendritic plate is plotted against *σ*. Solid line, stimulated synapse; dashed line, neighboring unstimulated synapse.

Suppose that the set of PFs responsible for a certain movement in a given context projects onto the PC dendritic plane according to a Gaussian distribution (Figure 6A**),** with the synapse of interest at its center. The 1-s–averaged concentration of NO will be given as 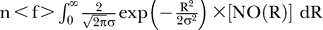 , where n and <f> are the total number of PFs that fire to generate the movement in that context, and their average firing rate, respectively. The product of n and <f> represents the activity of these PFs, and σ is the standard deviation of their spatial distribution. [NO(R)] is the 1-s–averaged concentration of NO that is synthesized in a single PF and measured at a distance of R μm (Figure 2D–2F). The function inside the integral sign, 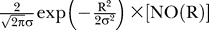 , represents the probability-weighed time-averaged concentration of NO derived from a PF responsible for the movement, because 
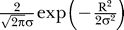
 denotes the probability density of such a PF occurring at distance R μm. This function, plotted against R in Figure 6B, clearly indicates that regardless of the size of *σ,* NO cannot be provided by PFs more than several dozens of micrometers away because of the restricted NO distribution (Figure 2F). Figure 6C plots *σ* against the n<f> at which [NO] reaches the EC_50_ (that is to say, the [NO] to phosphorylate [P-AMPAR]_50%,19min_ of AMPARs at 19 min) either for a stimulated synapse or for an adjacent unstimulated synapse. The interpretation is as follows: below the lower curve, no substantial LTD occurs in any synapse. Above the upper curve, LTD occurs not only in stimulated synapses, but also in neighboring synapses that are not directly stimulated, and synapse specificity is lost. Between the two curves, LTD is synapse-specific.

In reality, however, NO does not travel very far, and only PFs in the vicinity contribute to the local concentration of NO, regardless of the size of *σ* (Figures 2F and 6B). Thus, it must be much more practical to estimate the number and distribution of PFs that actually contribute to [NO] at the synapse of interest. Suppose that among the n PFs responsible for a given movement, n_90%_ within R_90%_ μm produce 90% of the EC_50_ of NO. R_90%_ and n_90%_ appear to represent the actual distribution and number of responsible PFs. Figure 6D plots *σ* against R_90%_, which was obtained by numerically solving 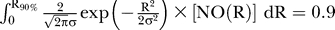 . R_90%_ reached a plateau at approximately 7 μm, suggesting that regardless of the size of *σ,* the state of a PF–PC synapse can be influenced only by PFs in the immediate vicinity. In Figure 6E, *σ* is plotted against n_90%_<f> for a stimulated synapse and a neighboring synapse not directly stimulated. In contrast to n<f>, as *σ* rose, n_90%_<f> plateaued at approximately 8 for the stimulated synapse and at 14 for the neighboring synapse. This implies that for any movement in any given context, the number of firing PFs in the vicinity needs to be strictly limited; otherwise, input specificity would be lost. Furthermore, regardless of the size of *σ,* AMPAR phosphorylation in a stimulated synapse in the intermediate phase was not achieved by the firing of a single PF, but by the firing of multiple PFs (Figure 6E). Thus, it can be concluded that plasticity of a synapse is regulated by the surrounding PF activity.

The ratio of n_90%_<f> to the total number of the PFs projecting within R_90%_ μm of the dendritic plate (Figure 6F) can be used as an index of the sparseness of predicted PF activity, and it will enable an easy comparison with our prediction with future experiments. The ratio is obtained from the following reckoning: the size of the PC dendritic plate is 250 μm × 300 μm, and 400,000 PFs traverse the dendritic arborization of a PC in cats [5]. Thus, the density of PFs projecting on the PC dendritic plate is estimated as 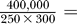 5.3/μm^2^, and the total number of PFs traversing a circle (of radius R_90%_ μm) on the dendritic plate is estimated as 
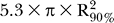
 . Figure 6F shows the ratio for the stimulated synapse, and that for the neighboring synapse, plotted against *σ*. Except at very small *σ* (≤2.3 μm), the ratios were less than 0.1 and approached 0.017 (at the stimulated synapse) and 0.0099 (at the neighboring synapse) as *σ* rose, suggesting that if LTD is input-specific in vivo, only several percent of PFs in the vicinity fire to encode a particular motion in any given context.

## Discussion

We simulated the sequence of events in cerebellar LTD from receptor activation to AMPAR phosphorylation in search of the cellular substrates of context-dependent motor learning [1,9,10,12]. The cellular events modeled here are not, of course, the whole picture of cerebellar LTD. For example, our model does not consider *δ*2 receptors [13], endocannabinoid signaling [44], dependence of NO release on the frequency of PF activity [35], mGluR-linked slow excitatory postsynaptic potentials [45], desensitization of soluble guanylyl cyclase [46], cyclic guanosine monophosphate (cGMP)–mediated activation of phosphodiesterase [47], gene expression, or protein synthesis [39,40]. However, this rather simple model was able to demonstrate the key features of PF–PC LTD (Figure 4) and shed light on some important characteristics of cerebellar LTD and learning (Figures 5 and 6).

The simulation results revealed that synapse-specific modification is theoretically possible despite the difficulty in demonstrating it experimentally [38]. Furthermore, [P-AMPAR] in the intermediate phase was found to be dependent on NO derived from surrounding PFs. During low PF activity in the vicinity, stimulated PF–PC synapses were incapable of undergoing prolonged AMPAR phosphorylation because of an insufficient NO concentration; during excessive PF activity, even neighboring synapses underwent AMPAR phosphorylation, and synapse specificity was lost. Only at a moderate level of PF activity in the vicinity did AMPAR phosphorylation occur in a synapse-specific manner. We predict that any movement in any context is encoded by a small number of PFs, because otherwise synapse specificity would be lost.

Although LTD can be induced by a large increase in calcium alone [48], in vivo studies have shown the essential role of NO in cerebellar learning and adaptation [13,49–51], suggesting that LTD in such a condition is unphysiological. Similarly, LTD induced by strong activation of PFs alone [42] can be regarded as another unphysiological extreme, because the predicted sparse activity of PFs in vivo (Figure 6) will never be sufficient for supralinear increase in calcium levels if it is not paired with CF firing. Between these two extremes lies our simulation setting, where LTD is induced by the increase of NO and calcium levels due to conjunctive activation of PFs and the CF, which corresponds better to the physiology of behaving animals.

### Synaptic Specificity

Our simulation shows that synapse-specific AMPAR phosphorylation is theoretically possible (Figure 4). Some slice experiments have demonstrated that, in most cases, standard procedures to induce homosynaptic PF–PC LTD also induce heterosynaptic LTD in neighboring synapses, bringing into question the input specificity of cerebellar LTD [38,41,42]. There are, however, strong reasons to expect input specificity in vivo, if not in vitro. Cerebellar LTD needs to be input-specific if it is the cellular substrate of supervised learning, as suggested experimentally and theoretically [13]. In addition, synapse-specific modification is more advantageous by orders of magnitude than the nonspecific form in terms of metabolic costs and memory capacity [13,41]. Moreover, neurons have acquired through evolution the apparatus to localize modification in individual spines, such as the intraspinal calcium store [13] and the calcium-compartmentalizing spine neck [52]. Thus, our demonstration of room for synapse-specific AMPAR phosphorylation is not at all trivial. The range of the number of stimulated synapses for input-specific LTD was found to be narrow (Figures 4D and S3), which accounts for the difficulty in demonstrating this form of LTD experimentally [38].

In contrast with the previous report that multiple PFs need to be stimulated in order to induce LTD [38], Casado et al. [19] demonstrated LTD at the synapse between a single granule cell (GC) and a PC. However, as is suggested in their paper [19], not only the PF–PC synapse, but also multiple synapses at the ascending part of the GC axon, could be involved. Further in vitro and in vivo studies are necessary to confirm the possibility that stimuli to a single synapse is sufficient for induction of LTD.

### NO Represents the Relevance of Contexts

**Figure 7:**
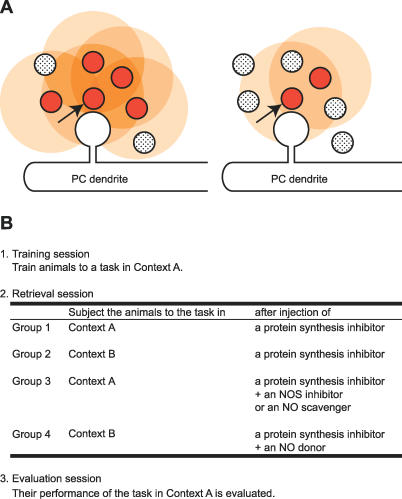
A Novel Hypothesis of the Cellular Mechanism for Context-Dependent Cerebellar Learning and an Experiment to Test It (A) Schematically shown are a PF–PC synapse and its surrounding PFs in a parasagittal section of the cerebellar cortex. Firing PFs are indicated by red solid circles, and those at rest are indicated by gray dotted circles (note that their beams are perpendicular to the sagittal plane.) Some neighboring PFs might fire together with the PF making the synapse (arrow) and produce NO (orange semitransparent circles); others might not. Left: when a few PFs in the neighborhood fire together, that will result in a sufficient concentration of NO for the PF–PC synapse to be modified. Right: when only a small number of PFs fire together, [NO] will not increase to the level necessary for the synapse to be modified. (B) The experimental protocol.

The IP_3_R, which is sensitive to both calcium and IP_3_, appears to be sufficient for the coincidence detection mechanism triggered by conjunctive stimulation of the PF and the climbing fiber (CF) [25,36]. So, what can NO add in terms of computation? Considering that only a fraction of PFs traversing a PC dendritic plate make electrical connections with the PC [43], this diffusing messenger seems to have a specific role in cerebellar learning. According to the results of our simulation, input-specific LTD necessitates that NO be provided by a modest number of surrounding PFs. This is a very important finding, because it might underlie context-dependent learning in the cerebellum [1,3,4,9,10], as we propose here. In our hypothesis, NO represents the relevance of a given context and enables context-dependent selection of internal models to be updated. Imagine that a PF–PC synapse responsible for a certain movement in a certain context is surrounded by thousands of PFs coding vast modalities of information, such as joint angles, audiovisual cues, verbal instructions, etc. The synapse will be modified only when it and a few of its surrounding PFs fire together and produce a sufficient concentration of NO (Figure 7A, left). However, if a small fraction of them happen to fire in an inappropriate situation, the synapse will remain unmodified, because [NO] does not reach the level necessary for prolonged AMPAR phosphorylation (Figure 7A, right). As a result, the internal models for a movement are acquired or updated only when they are responsible in the given context. In other contexts, where these internal models are not responsible, they are left unmodified. This corresponds to the system-level findings that the cerebellum can form modules of internal models according to the context [3,4], while adaptation to multiple tasks is interfered with in the absence of adequate contexts [8,12]. In a later subsection, we will propose an animal experiment to critically test our hypothesis (Figure 7B).

According to the Marr–Albus model [53,54], divergence of MF inputs to an enormous number of GCs enables pattern separation, so that after learning, similar sensory inputs are associated with different motor outputs in different contexts. However, a recent in vivo study revealed that a burst of activity in an MF is not filtered at the MF–GC synapse, but rather directly transmitted to the downstream GC [55], implying that input patterns are not likely to be separated there. Our hypothesis might provide a resolution by suggesting that pattern separation, or assignment of adequate internal models for a movement to be learned in a given context, takes place in the molecular layer instead, according to the [NO] determined by the surrounding PF activity in that context.

Because bursts of PF activity are more efficient than single pulses in induction of LTD in cerebellar slices [52,56], one may argue the possibility that repetitive activation of a single PF is sufficient for induction of LTD. However, it is very difficult to experimentally test this, because even the weakest stimuli might activate several PFs [36]. Bursts of PF activation might be too strong and less appropriate than single stimuli as an analog for physiological activation of PFs in vivo for the following reason: single activation of PFs in slices elicits repetitive firing through the antidromic activation of GCs [57], just like physiological stimuli in vivo typically evoke triplets of action potentials in GCs [55], presumably firing their PFs as many times. On the other hand, bursts of activation applied in slice experiments usually consist of five pulses, which are much greater stimuli than triplets in vivo, considering the multiplication of pulses in vitro [57].

A recent study implicated the presynaptic terminals of interneurons rather than PFs as the origin of NO [58]. If so, our simulation model and hypothesis would still apply, because the activity of interneurons reflects that of connected PFs, and their connection with multiple PFs might serve to integrate PF activity in the neighborhood. Namiki et al. [35] estimated [NO] after stimulation of PFs to be at micromolar levels, whereas Hall and Garthwaite estimated the peak concentration of NO produced by a single bouton to be 15 pM and the tissue concentration of NO to be 50 nM at most following full activation of NOS [59]. This severe discrepancy might reflect a lack of knowledge and understanding of NO kinetics in the brain. The peak concentrations of NO in our simulation lie between Hall's and Namiki's estimates, at a nanomolar level (Figure 2). Because we estimated a production rate of NO (k_f_[NOS]_total_) at which NO would mediate the spread of LTD at a realistic size of PF stimulation, our model as a whole yields quantitatively compatible outcomes with experimental findings in terms of AMPAR phosphorylation and LTD.

### Sparse PF Activity Codes a Movement in a Context

According to the results of our simulation, synapse specificity requires that every movement in every context be coded by a small number of PFs (Figure 6). This is in agreement with Marr and Albus' postulation that only a small fraction of PFs is active at any given time [53,54], and might at least partly explain the extreme difficulty in demonstrating in vivo PF activity induced by natural stimuli, in spite of recent technical developments in the bioimaging of and electrophysiology of the cerebellar cortex [55,60]. Sparse coding is also suggested in the hippocampus and cerebral cortices [61–63] and might be a general principle of information processing in the central nervous system.

The low PF activity estimated in this study does not contradict the high frequency of simple spikes (SSs) at approximately 50–100 Hz [5], because PCs intrinsically generate spontaneous spiking activity even in the absence of PF inputs [64]. Moreover, a simulation study [65] has shown that a slight increase in the PF firing rate can raise the SS frequency dramatically. Indeed, a burst of activity in only a few PF–PC synapses is sufficient to evoke large excitatory postsynaptic potentials in the soma. Assuming a maximum conductance of 1 nS at the spine and an input resistance of 200 MΩ at the soma (counterintuitively, this is at a level comparable with that of dendritic terminals [27]), and considering the relatively low electrical resistance of the spine neck and the short electrical length of the dendrite [24], a rough calculation shows that full activation of the AMPARs at a single spine can elevate the somatic potential by 10 mV. Thus, bursts of activity in several PFs only are probably sufficient to generate action potentials in a PC, increasing its SS rate.

### Proposal for an Animal Experiment

We propose an animal experiment to test our novel hypothesis that [NO] represents contextual relevance and enables context-dependent selection of internal models to be learned (Figure 7). Retrieval of a memory has been shown to make the memory labile in paradigms such as contextual fear conditioning and eyelid conditioning [66–68]. In other words, after retrieval, a new round of protein synthesis is necessary to preserve the memory in the future. In our proposed experiment, we take advantage of this retrieval-triggered transition of memory from a stable state to a labile state. First, animals are trained to perform a task in a particular context, Context A (wallpaper, odor, etc.), until their performance of the task reaches a plateau (training session). The task could be, for example, wheel running. The animals are then divided into four groups. Group 1 animals are subjected to the task again in Context A after injection of a protein synthesis inhibitor (retrieval session); Group 2 animals also perform the task after administration of the drug, but in a distinct context, Context B, instead of Context A. Their performance of the task in Context A is evaluated later (evaluation session). Considering a context-dependent modular organization of internal models in the cerebellum [3,4], it is expected in the evaluation session that Group 1 animals would show poor performance because the internal models for the task have been activated by the retrieval and prevented from reconsolidation by the protein synthesis inhibitor.

In contrast, Group 2 animals would show better performance because the internal models previously acquired in Context A are not fully activated during the retrieval in Context B and remain stable. This experiment will advance our understanding of cerebellar learning by pharmacologically proving context-dependent activation of internal models, which has been demonstrated only indirectly by functional imaging [3,4]. Our proposal for the experiment goes beyond that. In the retrieval session, Group 3 animals are subjected to the task in Context A after an NOS inhibitor or an NO scavenger is administered together with the protein synthesis inhibitor; Group 4 animals are subjected to the task in Context B after injection of a NO donor and the protein synthesis inhibitor. The expected performance of the task in the evaluation session would be as follows: Group 3 animals would be expected to perform better than Group 1 animals, because in the retrieval session the memory of the task in Context A would remain stable due to the shortness of NO. Group 4 animals would show poorer performance than Group 2 animals, because exogenous NO would perturb the context-dependent regulation of plasticity and make labile even those synapses to which Context B is not relevant.

In summary, this simulation study reveals that AMPAR phosphorylation at a synapse depends on [NO] determined by nearby PF activity, and this might form the foundation of context-dependent learning in the cerebellum. We predict that any movement in any context is coded by a small number of PFs, because otherwise input specificity would be lost. Lastly, we have proposed an animal experiment to critically test our hypothesis that NO represents the relevance of a given context and enables context-dependent selection of internal models to be updated.

## Materials and Methods

### Modeling a PC dendrite.

Our model is basically a PC dendrite represented as a passive electrical cable with spine compartments attached to it (Figure 1A). Resistance and capacitance of the plasma membrane, intracellular resistivity, and the parameters for ion channels are identical to the values used in De Schutter's model [24] unless mentioned otherwise, and are listed in their paper. When computing the electrical potential, the diameter of the cable was multiplied by the rescaling factor, rf ( = 9) and its length by the square root of rf so that the total membrane resistance and capacitance matched those of real PCs [27]. This multiplication does not alter the electronic length of the dendrite, because the space constant of a cable is proportional to the square root of its diameter [30]. We used the original size of the dendrite when computing diffusion of NO. Each spine was composed of a single-compartmental prolate spheroid whose major and minor axes were 0.58 μm and 0.49 μm long, respectively [28], connected to the dendrite by a spine neck. As calcium has been shown experimentally and theoretically to be compartmentalized in dendritic spines [52], we regarded the spine neck to be an electronic resistance of 5.1 × 10^7^ Ω (length, 0.58 μm [28]; diameter, 0.19 μm [28]; intracellular resistivity, 250 Ω • cm [24]) and did not consider the flow of ions through it. AMPAR currents were described as follows:

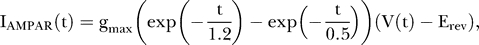
where t, g_max_, V, and E_rev_ are time after stimulation, maximum conductance of the AMPAR current (3.5 mS/cm^2^), the electrical potential of the spine, and the reversal potential of the AMPAR current (0 mV), respectively. The peak conductance of the currents equals the value obtained from a previous report [27]. Spines contained the P-type Ca^2+^ channel and the BK Ca^2+^-activated K^+^ channel described in Hodgkin–Huxley type equations [24]. The other ion channels in De Schutter's model were omitted because they are relatively rare in the distal dendrite, whose PF–PC synapses were of interest to us. As the P-type Ca^2+^ channel is localized at the spine head and rarely occurs in the dendritic shaft [69], we concluded that our model, a passive cable with spines containing ion channels, was a fairly accurate model of the PC dendrite. The potassium and calcium currents followed Ohm's law and the Goldman–Hodgkin–Katz current equation, respectively [70]. The maximum membrane permeability (p_max_) of the P-type Ca^2+^ channel to calcium was estimated to be 1.2 × 10^−13^ cm^3^/s per spine based on immunoelectron-microscopic studies that provided its unitary conductance [71,72] and the number of the channels per spine in the PF–PC synapse [69]. The increase in [Ca^2+^]_spine_ opened BK Ca^2+^-sensitive K^+^ channels as in De Schutter's model and repolarized the spine. Change in the electrical potential of spines was free to propagate through the dendrite and affect the potential of neighboring spines. The spines were also equipped with calcium buffers and pumps. Doi's model [25] provides their kinetics and parameter values. The model has shown that IP_3_Rs detect the coincidence of PF and CF inputs and release a large amount of calcium, and has predicted the maximum [Ca^2+^]_spine_ to be several micromolars and its peak to occur about 200 ms after stimulation. These values are quantitatively consistent with the results of in vitro experiments [36]. The IP_3_R (Figure 1B) detects the coincidence of PF inputs and depolarization and activates a large release of calcium from the ER [25]. For simplicity, our model postulates that during the 2 s after each stimulus, a fixed number (5.3 × 10^4^) of calcium ions flow from the ER into the coincidently stimulated spines (Figure S1), instead of simulating the mGluR–IP_3_R pathway.

### Kinetics of Nitric Oxide.

[NO] at various distances and times was obtained (Figure 2) by numerically solving the following differential equation:

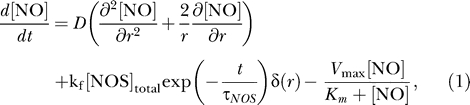
where *r* and *D* are distance between the bouton and the observation point in micrometers and the diffusion constant at 3.3 μm^2^/ms [23], respectively; and *δ* represents the Dirac's delta function, (that is, NO is produced only at the bouton). We modeled NOS as a Michaelis–Menten enzyme that was instantaneously activated at *t* = 0 (ms) and inactivated exponentially in a rather short time (time constant *τ_NOS_* = 50 ms), instead of actually simulating the complex mechanism of NOS activation [73]. Decay of NO also followed Michaelis–Menten kinetics (*K_m_* = 10 nM, *V_max_* = 1 μM/s) [59]. A parameter analysis revealed that the time-averaged NO concentration, which controlled AMPAR phosphorylation (Figure 5), was independent of the estimated parameter, *τ_NOS_* (Figure S2). Although *N*-methyl-D-aspartate receptors are thought to be implicated in NOS activation [73,74], single stimuli to PFs can induce NO synthesis and PF–PC LTD [38,42] for unknown reasons, as we assumed here. The distance between the presynaptic site of NO synthesis and its opposing spine was 1 μm in the model.

We estimated the unknown parameter, k_f_[NOS]_total_, in the following way: LTD has been reported to spread to neighboring PF–PC synapses when dozens of PFs are stimulated in synchrony with somatic depolarization [38,41], and the spread is NO-dependent [42]. According to another experiment [75], uncaged NO paired with somatic depolarization induces LTD, indicating that an increase in [Ca^2+^]_spine_ at submicromolar levels is sufficient for cerebellar LTD, if [NO] is high enough. Under the constraints of these findings, we chose to use k_f_[NOS]_total_ of 20 μM/s, at which [P-AMPAR]_50%,19min_ of AMPARs were phosphorylated at 19 min in synapses that were not directly stimulated by stimulation of 19 synapses (Figure 4D). Important results of our simulation—that AMPAR phosphorylation in the intermediate phase was not induced by stimulation of a single PF but of multiple PFs, and that input-specific LTD was actually possible (Figure 4)—were robust with respect to the k_f_[NOS]_total_ value (≤80 μM) (Figure S3), as indicated by the distance between N_50%_ for stimulated synapse (solid line) and that for its neighboring synapse (dotted line). At larger k_f_[NOS]_total_ values (≥120 μM), where the two curves almost converged, homosynaptic LTD was induced by stimuli to a single PF, but at the same time heterosynaptic LTD was also about to occur, and input specificity was almost ruined.

### Intracellular signaling cascades.

The signaling cascade in our model (Figure 1C) is based on Kuroda's model and uses the same parameter values that are listed in their paper and its supplementary information [26]. Kuroda's model demonstrates that PKC is activated by a linear pathway and by a positive feedback loop in the presence of NO. These signaling cascades are reviewed elsewhere in detail [13,26,76]. Briefly, NO diffusing from presynaptic terminals activates soluble guanylyl cyclase, which catalyzes the conversion of guanosine triphosphate to cGMP. cGMP activates cGMP-dependent protein kinase, which phosphorylates the G substrate, and the phosphorylated G substrate eventually inhibits protein phosphatase 2A [77], which then dephosphorylates MAP/extracellular signal-regulated kinase (MEK) and AMPARs. Calcium ions, either released from the ER or flowing in through the VGCC, activate cPLA2, resulting in the production of arachidonic acid and subsequent activation of PKC. In addition to arachidonic acid, Ca^2+^ and diacylglycerol also activate PKC. PKC activates the MAPK cascade, which consists of Raf, MEK, MAPK, and the MAPK phosphorylate cPLA2, forming a positive feedback loop.

However, we had to make several alterations to Kuroda's model because it was inconsistent with other parts of our model and previous findings in terms of calcium concentration. More specifically, (1) Kuroda's model assumes the basal [Ca^2+^]_spine_ to be 0 μM, whereas in reality it is about 0.05 μM [36], a value that is assumed in Doi's model [25]. This disagreement prevented the direct import of [Ca^2+^]_spine_ predicted in the dendritic part of our model. (2) Kuroda's model also assumes the peak [Ca^2+^]_spine_ of conjunctively stimulated synapses to be 0.55 μM, which is much smaller than the value estimated in Doi's model and in an experimental study [25,36].

Some modification was necessary to overcome these problems. Specifically, the dissociation constants of cPLA2 and PKC for calcium were reduced 100-fold, and the catalytic constants for the calcium-bound forms of these enzymes were reduced 11-fold and 2.7-fold, respectively. [Ca^2+^]_spine_ was imported to the signaling cascade part of the model (Figure 1C) from other parts that computed calcium influx, release, buffering, and pumping (Figure 1A and 1B) as (1) 0 μM when the predicted [Ca^2+^]_spine_ value was less than 0.05 μM, and as (2) the predicted [Ca^2+^]_spine_ value minus 0.05 μM, when the predicted value was 0.05 μM or greater.

The Lyn pathway and the CHRH pathway, which play only minor roles in Kuroda's model, were omitted in our model.

Despite these alterations, the behavior of our model was very similar to that of Kuroda's model, such as the time courses of activation of kinases and phosphorylation of the receptors. Stimulation of a few synapses (11, 20, or 29) led to activation of MAPK (Figure S4A) and PKC (Figure S4B), and to phosphorylation of AMPARs (Figure 4A and 4B) during the intermediate phase; the time courses for these events were quantitatively similar to those in Kuroda's model. In addition, phosphorylation of AMPARs followed the all-or-none principle in our simulation (Figure 4), as in Kuroda's model (see Figure 4 of [26]). We stimulated synapses for various durations and measured [P-AMPAR] at 19 min, as plotted against the duration of stimulation in Figure S4C. AMPAR phosphorylation after stimulation of 11, 20, and 29 synapses clearly demonstrated the all-or-none principle according to the duration of stimulation, and the optimal duration of stimulation that induced stable phosphorylation of AMPARs in the intermediate phase was comparable to that predicted by Kuroda's model.

### Stimulation conditions.

The neighboring synapses to be stimulated were chosen so that they roughly followed a Gaussian distribution with a standard deviation of 5 μm to mimic stimuli applied with a thin electrode (Table S1). The synapse of interest (9 μm from the tip of the dendrite) was at the center of the Gaussian distribution. The soma, which was actually the proximal end of the dendrite, was depolarized from −70 mV to 0 mV for 100 ms in synchrony with the synaptic stimuli. Thus, all synapses on the dendrite were either conjunctively stimulated (comprising PF–PC stimulation and depolarization of the soma), or only depolarized without direct application of stimuli to the synapses.

In this study, single stimulation of PF–PC synapses was used instead of PF bursts. Actually, single stimulation of PFs in vitro can be regarded as an analog for their bursts in vivo (Discussion), and it is the bursts that convey information in the cerebellum. Accumulating evidence suggests that spontaneous activity of MFs and GCs is irrelevant in terms of cerebellar computation, and that information in the MF–GC–PC pathway is transmitted in the form of bursts. Spontaneous MF inputs in anesthetized rats do not evoke action potentials in GCs [55]. GCs also fire spontaneously at 0.5 Hz [55], and hundreds of thousands of them project onto each PC via PFs [5]. Thus, a PC is estimated to receive several dozens of thousands of PF inputs per second in the resting state. Microinjection of an AMPAR antagonist, however, has no effect on the spontaneous firing rate in the majority of PCs [64], implying that spontaneous firing of MFs and GCs does not contribute to generation of SSs in PCs, and that PCs intrinsically generate spontaneous spiking activity in vivo as well as in vitro [78]. Furthermore, spontaneous activity in GCs is also likely to have only a negligible effect on the production of NO in PFs, because it requires repetitive firing at a high frequency, such as dozens of pulses at 20 Hz [18]. By contrast, sensory stimulation produces bursts of MF excitatory postsynaptic currents, which then successfully trigger bursts of spikes in GCs [55]. A burst of activity in the GC layer is associated with SSs in the PCs [79]. Thus, it is highly likely that sensory information is transmitted from MFs to GCs and PCs in the form of bursts, and that their spontaneous activity is filtered out at MF–GC and PF–PC synapses. Paired-pulse facilitation [13,57] might explain the strong preference of PCs for PF bursts. The evidence mentioned above suggests that only bursts can produce NO and transmit information over synapses in vivo.

### Robustness of the model.

Although most parameters were available from experimental studies and existing simulation models [23–28,65,67,69,71,72], we still had to estimate some of them. We evaluated the robustness of our model with respect to these newly estimated parameters, including the surface area of the dendrite, p_max_ of the VGCC, g_max_ of the BK channel, and the amount of calcium released by the ER (Figure S5).

## Supporting Information

Figure S1 Calcium Release by the Endoplasmic Reticulum The stereotypical release in the model is shown.
(337 KB EPS)

Figure S2 Kinetics of Nitric Oxide at Various *τ_NOS_* Values In the left-hand panels, [NO] is plotted against time and distance; in the right-hand panels, the time-averaged concentration of NO is plotted against distance. [NO] is normalized so that the time-averaged concentration of NO at a distance of 1 μm equals 1 (arbitrary unit). [NO] at distances smaller than 0.5 μm are not shown, because the delta function in Equation 1 made the [NO] near the site of synthesis dependent on the discretization size of *r* in its numerical solution. *τ_NOS_* = 0.005 s (A), 0.05 s (B), 0.5 s (C), or 5 s (D). This figure demonstrates various lifespans of NO according to *τ_NOS_*, but clearly indicates that the time-averaged spatial distribution of NO is independent of *τ_NOS_*.
(438 KB EPS)

Figure S3 N_50%_ at Various k_f_[NOS]_total_ Values N_50%_ (see “Time Course of AMPAR Phosphorylation” in the Results section for a definition) in a stimulated synapse (solid line) and in a neighboring synapse (dashed line) are plotted against k_f_[NOS]_total_. The closed circles indicate the outcomes at the k_f_[NOS]_total_ value used.
(286 KB EPS)

Figure S4 Similarity with Kuroda's Model (A,B) Simulated time courses of the concentrations of active MAPK and active PKC. Bold lines under the plots indicate the duration of stimulation (1 Hz, 5 min).
(C) The duration of stimulation was parameterized between 0 min and 15 min, and the [P-AMPAR] at 19 min is plotted. Two (blue solid line), 11 (red dotted line), 20 (black dashed line), or 29 synapses (green dash-dotted line) were stimulated.
(348 KB EPS)

Figure S5 Robustness of Our Model with Respect to Estimated Parameters One of the most important characteristics of our model is the demonstration of AMPAR phosphorylation that is synapse-specific and at the same time dependent on the surrounding PF activity. The N_50%_ ratio (the ratio of N_50%_ for a stimulated synapse to that for an adjacent unstimulated synapse) reflects the range of inputs that achieve synapse-specific LTD, and it is expected to remain constant when estimated parameters were varied. The N_50%_ ratio at 19 min is plotted against (A) the rescaling factor, rf*,* (B) the p_max_ for the P-type Ca^2+^ channel, (C) the g_max_ for the BK-type K^+^ channel, and (D) the amount of calcium released by the ER. The closed circles represent the outcomes obtained at the parameter value used.
(A) The rescaling factor rf. When rf was parameterized, the N_50%_ ratio remained almost constant, indicating that the model was robust against change in rf.
(B) p_max_ for the P-type VGCC. p_max_ was varied between 1/10-fold and 10-fold of the estimated value, 1.2 × 10^−13^ cm^3^/s/spine. The N50% ratio was very sensitive to changes in the p_max_ value. This is at least partly because we assumed a fixed number of calcium ions to be released from the ER upon stimulation, instead of actually simulating the mGluR and IP3 pathway. If our model had simulated the IP_3_ receptor pathway as theirs did, p_max_ values smaller than the estimated value would have led to the following consequences: depolarization would have introduced a smaller amount of calcium into the spine through the P-type calcium channels; IP_3_ receptors would have been less activated; fewer calcium ions would have been mobilized into the cytoplasm; and PKC and the MAPK-mediated positive feedback loop would have been activated to a lesser degree, leaving more AMPARs unphosphorylated in neighboring synapses, and making the N50% ratio smaller than the estimate in our model. On the other hand, p_max_ values greater than the estimated value yielded smaller N50% ratios because depolarization of the soma brought a sufficient amount of Ca^2+^ into the spine through P-type calcium channels to activate the MAPK-mediated positive feedback loop.
(C) g_max_ of the BK-type K^+^ channel. The ratio was very robust to change in the g_max_ value when it was varied between 1/10-fold and 10-fold of the original value, 80 mS/cm^2^.
(D) Amount of calcium released by the ER. This parameter was varied between 1/4-fold and 4-fold of the estimate (5.3 × 10^4^ ions). The N_50%_ ratio was very robust against changes in this parameter.
(305 KB EPS)

Table S1 Synapses To Be Stimulated (28 KB XLS)

## Abbreviations

AMPAR: alpha-amino-3-hydroxy-5-methyl-4-isoxazole propionate receptor
[Ca^2+^]_spine_: concentration of calcium in the spine
cGMP: cyclic guanosine monophosphate
cPLA2: cytosolic phospholipase A2
ER: endoplasmic reticulum
GC: granule cell
IP3: inositol 1,4,5-triphosphate
IP3R: inositol 1,4,5-triphosphate receptor
LTD: long-term depression
MAPK: mitogen-activated protein kinase
MEK: MAP/extracellular signal-regulated kinase
MF: mossy fiber
mGluR: metabotropic glutamate receptor
N: number of stimulated synapses
NO: nitric oxide
[NO]: concentration of nitric oxide
NOS: nitric oxide synthase
[P-AMPAR]: concentration of phosphorylated AMPARs
PC: Purkinje cell
PF: parallel fiber
PKC: protein kinase C
SF: sloping factor
SS: simple spike
VGCC: voltage-gated calcium channel
